# Efficacy and safety of equine anti-thymocyte immunoglobulin (eATG) in three Japanese patients with moderate to very severe aplastic anemia: a case series

**DOI:** 10.1007/s12185-022-03496-5

**Published:** 2022-11-28

**Authors:** Yoshinobu Kanda, Takehiko Mori, Atsushi Narita, Kevin D. Wolter, Hiroki Yoshimatsu, Kazuma Nishimura

**Affiliations:** 1 Division of Hematology, Department of Medicine, Jichi Medical University, 3311-1 Yakushiji, Shimotsuke-Shi, Tochigi-Ken, 329-0498 Japan; 2 Division of Hematology, Department of Medicine, Keio University School of Medicine, Tokyo, Japan; 3 Department of Hematology, Tokyo Medical and Dental University, Tokyo, Japan; 4 Department of Pediatrics, Nagoya University Graduate School of Medicine, Nagoya, Japan; 5 Pfizer Inc, New York, NY USA; 6 Pfizer R&D Japan, Tokyo, Japan

**Keywords:** Equine anti-thymocyte immunoglobulin, Aplastic anemia, Hematologic response, eATG, Safety

## Abstract

Aplastic anemia results from lymphocyte-mediated destruction of hematopoietic stem cells. Immunosuppressive therapy with anti-thymocyte globulin (ATG) and cyclosporine is the standard front-line treatment for patients with severe aplastic anemia who are not suitable candidates for stem cell transplants. PF-06462700 is a potent equine ATG that targets T-lymphocytes and has been approved as a treatment for aplastic anemia outside of Japan for over 30 years. Due to the high medical need for PF-06462700, the Ministry of Health, Labor and Welfare requested its development for Japanese patients with aplastic anemia. In this case series, the efficacy and safety of PF-06462700, administered intravenously at 40 mg/kg/day for 4 days, were assessed over a 24-week period. This was as an open-label, single-arm, multicenter clinical study designed to enroll a minimum of three Japanese participants with aplastic anemia. Two participants met the primary outcome of hematologic response at week 12 and improvements in disease severity were observed. No deaths or serious adverse events were reported. The efficacy results from this case series suggest that administration of PF-06462700 is generally well-tolerated and produces a hematologic response in Japanese patients with aplastic anemia, which should be further evaluated in real-world studies.

ClinicalTrials.gov identifier: NCT04350606.

**Supplementary Information:**

The online version contains supplementary material available at 10.1007/s12185-022-03496-5.

## Introduction

Aplastic anemia is mainly caused by immune-mediated destruction of hematopoietic stem cells by lymphocytes, resulting in pancytopenia [1]. The global incidence of aplastic anemia varies: between 2004 and 2012 there was an estimated incidence of 8.2 cases of aplastic anemia per 1,000,000 person-years in Japan [2], which is considerably higher than the estimated incidence of 2.34 cases per million person-years in a European population study [3]. Standard front-line treatments for aplastic anemia include hematopoietic stem cell transplantation [4] or immunosuppressive therapy with anti-thymocyte globulin (ATG) and methylprednisolone plus cyclosporine (the latter for patients who are not suitable candidates for stem cell transplants) [5]. As part of their treatment strategy, patients with aplastic anemia also receive crucial supportive care, which includes transfusions of red blood cells and platelets, and prophylaxis with antimicrobials to prevent potentially fatal infections [1].

Equine ATG (eATG; PF-06462700) is an immunosuppressant that targets T-lymphocytes and has been available for over 30 years outside of Japan for the treatment of moderate-to-severe aplastic anemia [6]. Rapid depletion of lymphocytes with PF-06462700 was observed in a previous clinical study in patients with aplastic anemia [7]. eATG had better efficacy as a first-line treatment for severe aplastic anemia than rabbit ATG (rATG), as demonstrated by a significantly higher hematologic response rate following treatment with eATG compared with rATG, which subsequently translated into a higher survival rate [7]. In preclinical studies, PF-06462700 also stimulated both the growth of erythroid progenitor cells and the release of hematopoietic growth factors in vitro [8, 9]. It is possible that these activities also contribute to the therapeutic effect of PF-06462700 in aplastic anemia [8].

In October 2018, the 36^th^ Meeting of the Study Group on Unapproved and Off-label Drugs of High Medical Need evaluated PF-06462700 as a drug with high medical need for aplastic anemia in Japan, and its development was requested by the Research and Development Division, Health Policy Bureau, Ministry of Health, Labor and Welfare in November 2018. PF-06462700 was designated as an Orphan Drug in Japan on March 11, 2021. A previous clinical study indicated the usefulness of two doses of PF-06462700 (10–20 mg/kg/day for 8 days) in 50 Japanese patients with aplastic anemia [10]; however, additional clinical data are required to support further development of PF-06462700 40 mg/kg/day for 4 days for use in Japan. This case series, conducted as an open-label, single-arm, multicenter clinical study, with design agreed by the Pharmaceuticals and Medical Devices Agency, assessed the efficacy and safety of PF-06462700 40 mg/kg/day for 4 days in Japanese patients with moderate and above aplastic anemia.

## Methods

### Participants

A minimum of three patients with aplastic anemia was planned for this non-statistically powered case series, based on the high medical need for PF-06462700 in Japan and the feasibility of the study in this rare disease, as agreed between the sponsor and the Pharmaceutical and Medical Devices Agency. Participants eligible for inclusion were males or females aged ≥ 2 years who had a clinical diagnosis of aplastic anemia based on either bone marrow aspiration and biopsy findings, magnetic resonance imaging, or both. Eligible participants also met criteria for moderate or above aplastic anemia (Stage 2b or worse) based on the diagnosis criteria established by the Japanese Study Group on Idiopathic Hematopoietic Disorders [11].

Exclusion criteria included: patients who were eligible and willing to have a sibling allogenic stem cell transplantation; evidence of a myelodysplastic syndrome (except for refractory cytopenia in children) or other primitive marrow diseases; history or suspicion of congenital aplastic anemia; history of malignant tumors with active disease within 5 years; infection with hepatitis B or C virus, human immunodeficiency virus, or human T-cell leukemia virus type 1; hypersensitivity after the skin test of the study drug; severe hepatic, renal, or cardiac failure, or any other life-threatening concurrent (aspartate aminotransferase, alanine aminotransferase, or total bilirubin values > 5 × upper limit of normal [ULN], and/or creatinine values > 2 × ULN); uncontrolled severe infection; vaccination with a live vaccine or live attenuated vaccine within 6 weeks of the first dose of study drug; prior stem cell transplant; or prior immunosuppressive therapy with lymphocyte-depleting agents/therapies, including both non-B-cell-selective and B-cell-depleting agents.

### Study design

Participants were enrolled across three sites in Japan between 2020 and 2021. Following a screening period of up to 4 weeks, patients received intravenous PF-06462700 (Pfizer Inc, Michigan, USA) at 40 mg/kg/day for 4 days, with a 24-week follow-up period. Infusion time ranged from 4 to 24 h. Concomitant use of granulocyte-colony stimulating factor, eltrombopag, romiplostim, and anabolic steroids was permitted only if necessary; however, lymphocyte-depleting agents, ATGs (other than study drug), and immunosuppressants (except for cyclosporine and corticosteroids), were not permitted. The study protocol was reviewed and approved by the Institutional Review Board and/or Independent Ethics Committee at each study site, and the study was registered with Clinicaltrials.gov (NCT04350606). The study was conducted in accordance with international guidelines, including the Declaration of Helsinki, the Council for International Organizations of Medical Sciences International Ethical Guidelines, and the International Council for Harmonisation Good Clinical Practice Guidelines, along with local laws and regulations. Signed and dated informed consent was obtained from each participant before any study-specific activity was performed.

### Efficacy outcomes

The primary endpoint was hematologic response at week 12, defined as meeting ≥ 2 of the following criteria (independently of administration of growth factors or transfusion): absolute neutrophil count ≥ 500/µL; platelet count ≥ 20,000/µL; and reticulocyte count ≥ 60,000/µL. Secondary efficacy endpoints were hematologic response at week 24; hematologic test values (absolute neutrophil, platelet, and reticulocyte counts) at day 4 and at weeks 1, 2, 4, 6, 8, 10, 12, and 24; survival status; and transfusion independence at weeks 12 and 24, based on absence of any record of transfusion after the first dose of PF-06462700 to the day of the week 12 visit (inclusive) for week 12, and from the day after the week 12 visit to the week 24 visit (inclusive) for week 24. For patients with aplastic anemia Stage 2b/3 at screening or baseline, an improvement in disease stage was also required to meet the hematologic response criteria.

### Safety outcomes

Safety was monitored throughout the study based on treatment-emergent adverse events (TEAEs), serious adverse events, adverse events leading to discontinuation, vital signs, physical examination, 12-lead electrocardiograms (ECG), and clinical safety laboratory assessments. Adverse events were classified according to Medical Dictionary for Regulatory Activities, version 24.0.

### Statistical analyses

Efficacy analyses were conducted on the full analysis set. The full analysis set and the safety analysis set included all participants who received ≥ 1 dose of PF-06462700. No statistical testing and inference were planned. Efficacy analyses were conducted without imputation for missing data, and all observations were included regardless of prohibited concomitant medication. Efficacy and safety data were reported for individual participants.

## Results

### Participants

Three Japanese participants aged 14–47 years, one male and two female with severe or very severe disease at baseline (Stage 4 or 5) were enrolled in this study. Prior to the start of the study, all three participants received transfusions of red blood cells and platelets. In addition, one participant had previously received medication for aplastic anemia rATG, cyclosporine, eltrombopag, and filgrastim. All three participants received concomitant cyclosporine and filgrastim and one participant received romiplostim during the study. All three participants enrolled in the study completed the treatment and follow-up periods.

### Efficacy

Two of the three participants achieved hematologic response at week 12 with PF-06462700, which was accompanied by improvements in disease severity from Stage 4 to Stage 1, and from Stage 5 to Stage 3 (Table 1). The remaining participant also met the defined response criteria for all cell types at week 12. However, the counts were deemed to be dependent upon the administration of romiplostim during the study; therefore, the participant’s hematologic response was classified as “not effective”. This participant demonstrated an improvement in disease severity from Stage 5 to Stage 1.

**Table 1:** Hematologic response at weeks 12 (primary endpoint) and 24 (full analysis set)

Subject	Hematologic response^a^	Severity of aplastic anemia (stage)			Absolute neutrophil count			Platelet count				Reticulocyte count		
		BL	Week 12	Week 24	(per μL)	Improvement depends on transfusion	Improvement depends on hematopoietic growth factor	(per μL)	Improvement depends on transfusion		Improvement depends on hematopoietic growth factor	(per μL)	Improvement depends on transfusion	Improvement depends on hematopoietic growth factor
Week 12														
1	Effective	4	1	–	2300	No	No	77,000	No		No	84,300	No	No
2	Effective	5	3	–	450	No	No	47,000	No		No	69,000	No	No
3	Not effective	5	1	–	5330	No	Yes	88,000	No		Yes	261,200	No	Yes
Week 24														
1	Effective	4	–	1	2700	No	No	139,000	No		No	53,760	No	No
2	Effective	5	–	1	750	No	No	58,000	No		No	85,000	No	No
3	Not effective	5	–	2A	1755	No	Yes	16,000	No		Yes	57,600	No	Yes

*BL* baseline

^a^Effective defined as meeting ≥ 2 of the following criteria: absolute neutrophil count ≥ 500/µL; platelet count ≥ 20,000/µL; reticulocyte count ≥ 60,000/µL. Improvement in counts that were dependent upon exogenously administered growth factors or transfusion were not considered as fulfilling response criteria. For subjects with Stage 2b or 3 at baseline/screening, hematologic response was defined as “effective” when 2 or more of the above-mentioned criteria were met and stage was improved

**Fig. 1:**
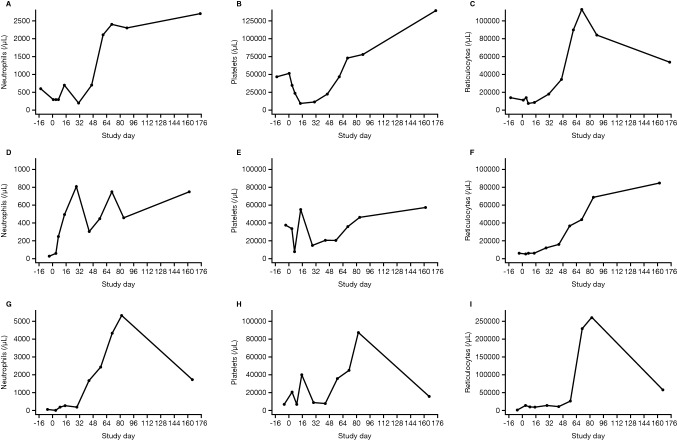
Hematologic cell counts for absolute neutrophils, platelets, and reticulocytes in individual study participants. Participant 1 (**A–C**), participant 2 (**D–F**), and participant 3 (**G–I**)

At week 24, the hematologic response was consistent with that observed at week 12 (Table 1). In addition, increases in absolute neutrophil, platelet, and reticulocyte counts from baseline through week 24 were observed in all three participants (Fig. 1). Following treatment with PF-06462700 40 mg/kg/day for 4 days, all three participants remained transfusion-dependent at week 12 (i.e., all participants required transfusion within the 12 weeks prior to evaluation). However, two of the participants were transfusion-independent at week 24. For all participants, the frequency of blood transfusions decreased over time (Supplementary material, Table S1).

### Safety

A total of 42 TEAEs were reported in the three participants. All three participants experienced ≥ 1 TEAE, most of which resolved during the study. The most common all-causality TEAEs observed in ≥ 2 participants were abdominal pain, nausea, hyperglycemia, and hypertension. All TEAEs were mild or moderate in severity. Moderate TEAEs were abdominal pain, nausea, hypogammaglobulinemia, staphylococcal infection, increased C-reactive protein, decreased oxygen saturation level, insomnia, and nail bed inflammation (each reported in one participant).

A total of 10 TEAEs were reported to be treatment-related, all of which were mild in severity. All three participants reported ≥ 1 treatment-related TEAE (Table 2). With regard to infectious complications, two participants developed cytomegalovirus (CMV) reactivation, with one reported as CMV infection, which was controlled by antivirals, and the other reported as CMV viremia, which did not require antiviral treatment. Decreases in white blood cells and lymphocytes were each reported once. There were no incidences of Epstein–Barr virus reactivation, deaths or serious TEAEs reported during the study. No clinically significant changes in laboratory parameters, vital signs, or ECG were reported.

**Table 2:** Overview of treatment-related TEAEs by MedDRA System Organ Class and Preferred Term (safety analysis set)

System organ class Preferred term	PF-06462700 40 mg/kg/day (*N* = 3)
Any treatment-related TEAE	3^a^
Gastrointestinal disorders	1
Abdominal pain	1
General disorders and administration site conditions	2
Feeling abnormal	1
Infusion site extravasation	1
Edema	1
Immune system disorders	1
Serum sickness	1
Infections and infestations	2
Cytomegalovirus infection	1
Cytomegalovirus viremia	1
Investigations	2
Blood creatinine increased	1
Lymphocyte count decreased	1
White blood cell decreased	1

Data shown are number of subjects with individual TEAE; subjects could have had more than one TEAE in the same System Organ Class

*MedDRA* medical dictionary for regulatory activities, version 24.0*, TEAE* treatment-emergent adverse event

^a^A total of 10 events were recorded

## Discussion

This case series, conducted as an open-label, single-arm, multicenter clinical study, assessed the efficacy and safety of PF-06462700 in Japanese patients with aplastic anemia. Although the number of enrolled participants was small, this was inline with anticipated recruitment numbers based on the rarity of aplastic anemia in Japan. Baseline disease characteristics and use of prior therapies were as expected for patients with severe aplastic anemia. All participants received concomitant immunosuppressive therapy with cyclosporine throughout the study participation period, which has been shown to improve response to ATGs [5] and is recommended in combination with ATG therapies in Japanese guidelines [11].

An effective hematologic response was documented for two of the three participants while improvements in stage of disease from baseline was observed in all three participants by week 12. Similar findings were reported in previous non-Japanese studies, with a hematologic response rate of 50–62% for 3 months and 50–68% at 6 months observed following treatment with 40 mg/kg/day of eATG for 4 days plus cyclosporine, in patients with severe aplastic anemia [7, 12]. In addition, a retrospective analysis of real-world use of eATG reported an overall response rate at 12 months of 69.2% following eATG treatment in patients with moderate-to-severe aplastic anemia [13].

Two participants were transfusion-independent by week 24, and there was an overall gradual reduction in the frequency of blood transfusions for all three participants. The participant who remained transfusion-dependent at week 24 had Stage 5 disease at baseline and did not meet the criteria for hematologic response at weeks 12 or 24. Despite cell counts meeting the criteria for “effective”, the investigator judged the response to be “not effective” in this participant, since these counts might have been related to administration of romiplostim, a thrombopoietin receptor-agonist that can stimulate erythrocyte, neutrophil, and platelet production in patients with aplastic anemia [14]. The same participant had previously received, and was refractory to, a combination of eltrombopag and rATG, and thus the efficacy of PF-06462700 as a second-line treatment was suggested but requires further investigation.

Our results suggest that intravenous administration of PF-06462700 at 40 mg/kg/day for 4 days is well-tolerated in Japanese patients, as no serious TEAEs or new safety signals were identified. Based on initial results from a previous clinical trial, PF-06462700 could be used in combination with the thrombopoietin receptor-agonist eltrombopag, as higher response rates were observed following treatment with standard immunosuppressive therapy (eATG and cyclosporine) and eltrombopag, compared with standard treatment alone [15].

Only three participants were enrolled in this case series. As planned, the results were not statistically verified, which may limit the ability of the study to draw definite conclusions. Hence, results should be interpreted with caution. A further limitation was the open-label, single-arm study design.

The results presented suggest that intravenous administration of PF-06462700 40 mg/kg/day is well-tolerated and produces an effective hematologic response in Japanese patients with aplastic anemia. The efficacy and safety of PF-06462700 in Japanese patients was comparable with findings in previous clinical studies in Western participants. Due to the small sample size, the efficacy and safety of PF-06462700 in Japanese patients should be further investigated in real-world studies with larger patient populations.

## Supplementary Information

Below is the link to the electronic supplementary material.

Supplementary file1 (DOCX 15 KB)

## Data Availability

Upon request, and subject to review, Pfizer will provide the data that support the findings of this study. Subject to certain criteria, conditions, and exceptions, Pfizer may also provide access to the related individual de-identified participant data. See https://www.pfizer.com/science/clinical-trials/trial-data-and-results for more information.
