# Correction: Efficacy and safety of equine anti-thymocyte immunoglobulin (eATG) in three Japanese patients with moderate to very severe aplastic anemia: a case series

**DOI:** 10.1007/s12185-023-03543-9

**Published:** 2023-01-25

**Authors:** Yoshinobu Kanda, Takehiko Mori, Atsushi Narita, Kevin D. Wolter, Hiroki Yoshimatsu, Kazuma Nishimura

**Affiliations:** 1 Division of Hematology, Department of Medicine, Jichi Medical University, 3311-1 Yakushiji, Shimotsuke-Shi, Tochigi-Ken, 329-0498 Japan; 2 Division of Hematology, Department of Medicine, Keio University School of Medicine, Tokyo, Japan; 3 Department of Hematology, Tokyo Medical and Dental University, Tokyo, Japan; 4 Department of Pediatrics, Nagoya University Graduate School of Medicine, Nagoya, Japan; 5 Pfizer Inc, New York, NY USA; 6 Pfizer R&D Japan, Tokyo, Japan

**Correction: International Journal of Hematology (2022) 117:37–43** 10.1007/s12185-022-03496-5.

The article “Efficacy and safety of equine anti-thymocyte immunoglobulin (eATG) in three Japanese patients with moderate to very severe aplastic anemia: a case series”, written by Yoshinobu Kanda, Takehiko Mori, Atsushi Narita, Kevin D. Wolter, Hiroki Yoshimatsu, Kazuma Nishimura, was originally published electronically on the publisher’s internet portal on 28 November 2022 without open access. With the author(s)’ decision to opt for Open Choice the copyright of the article changed on 16 January 2023 to © The Authors 2023 and the article is forthwith distributed under a Creative Commons Attribution 4.0 International License, which permits use, sharing, adaptation, distribution and reproduction in any medium or format, as long as you give appropriate credit to the original author(s) and the source, provide a link to the Creative Commons licence, and indicate if changes were made. The images or other third party material in this article are included in the article's Creative Commons licence, unless indicated otherwise in a credit line to the material. If material is not included in the article's Creative Commons licence and your intended use is not permitted by statutory regulation or exceeds the permitted use, you will need to obtain permission directly from the copyright holder. To view a copy of this licence, visit http://creativecommons.org/licenses/by/4.0/

