# Supplementary material for: Efficacy and safety of equine anti-thymocyte immunoglobulin (eATG) in three Japanese patients with moderate to very severe aplastic anemia: a case series
Source: Int J Hematol. 2022 Nov 28;117(1):37–43. doi: 10.1007/s12185-022-03496-5 (PMC9876848; doi:10.1007/s12185-022-03496-5)
Supplement: Supplementary file 1 — Supplementary file1 (DOCX 15 KB) [file 12185_2022_3496_MOESM1_ESM.docx]

# Supplementary material

**Table S1.** Number of blood transfusions by study participant across 4-weekly periods from baseline to Week 24 (safety analysis set)

| Subject | Blood cell type | Week -4 to BL | | BL to Week 4 | | Weeks 5 to 8 | | Weeks 9 to 12 | | Weeks 13 to 16 | | Weeks 17 to 20 | | Weeks 21 to 24 | |
| --- | --- | --- | --- | --- | --- | --- | --- | --- | --- | --- | --- | --- | --- | --- | --- |
|  |  | No. times | Total dose (U) | No. times | Total dose (U) | No. times | Total dose (U) | No. times | Total dose (U) | No. times | Total dose (U) | No. times | Total dose (U) | No. times | Total dose (U) |
| 1 | Platelets | 4 | 40 | 8 | 80 | 2 | 20 | 0 | 0 | 0 | 0 | 0 | 0 | 0 | 0 |
|  | Red blood cells | 4 | 8 | 3 | 6 | 2 | 4 | 0 | 0 | 0 | 0 | 0 | 0 | 0 | 0 |
| 2 | Platelets | 8 | 115 | 9 | 90 | 4 | 50 | 2 | 20 | 0 | 0 | 0 | 0 | 0 | 0 |
|  | Red blood cells | 4 | 8 | 4 | 8 | 2 | 4 | 3 | 6 | 0 | 0 | 0 | 0 | 0 | 0 |
| 3 | Platelets | 6 | 60 | 7 | 70 | 5 | 50 | 0 | 0 | 0 | 0 | 1 | 10 | 4 | 40 |
|  | Red blood cells | 3 | 6 | 4 | 8 | 3 | 6 | 1 | 2 | 0 | 0 | 0 | 0 | 0 | 0 |

*BL* baseline, *No* number of, *U* units
